# CastelLact Project: Exploring the Nutritional Status and Dietary Patterns of Pregnant and Lactating Women—A Comprehensive Evaluation of Dietary Adequacy

**DOI:** 10.3390/nu16162705

**Published:** 2024-08-14

**Authors:** Carmen I. Sáez Lleó, Carla Soler, Jose M. Soriano, Nadia San Onofre

**Affiliations:** 1 Food & Health Lab, Institute of Materials Science, University of Valencia, 46980 Paterna, Spain; saez_carlle@gva.es (C.I.S.L.); carla.soler@uv.es (C.S.); nsan_onofre@uoc.edu (N.S.O.); 2 Joint Research Unit on Endocrinology, Nutrition and Clinical Dietetics, University of Valencia-Health Research Institute La Fe, 46026 Valencia, Spain; 3 Department of Community Nursing, Preventive Medicine and Public Health and History of Science, University of Alicante, 03690 Alicante, Spain; 4 FoodLab Research Group, Faculty of Health Sciences, Universitat Oberta de Catalunya, Rambla del Poblenou 156, 08018 Barcelona, Spain

**Keywords:** breastfeeding, nutritional status, diet, pregnant women, health promotion

## Abstract

Promoting optimal nutrition in pregnant and lactating women is crucial for maternal and infant health. This study evaluated their nutritional status and dietary habits, assessing macro and micronutrient intake based on recommendations. A descriptive study with Spanish participants examined social, obstetric, dietary, and anthropometric data using quantitative and qualitative methods. The analysis of fatty acids by gas chromatography revealed significant variability, with notable deviations in specific fatty acids like C:10:0 and C:12:0. Despite some differences, the overall composition aligns with standards. During pregnancy, 53.8% consumed five meals/day. Grilling (92.3%) and baking (76.9%) were common. Food consumption frequency differed from recommendations. Lactating mothers’ mean energy intake was 2575.88 kcal/day ± 730.59 standard deviation (SD), with 45% from carbohydrates and 40% from lipids, including 37.16 g ± 10.43 of saturated fatty acids. Diets during pregnancy lacked fruits, vegetables, legumes, nuts, and cereals. Lactating mothers partially met nutritional objectives, with an energy distribution skewed towards lipids and deficiencies in calcium, iodine, vitamin D, E, and folic acid. Promoting proper nutrition during pregnancy and lactation is essential to safeguard health and prevent chronic diseases.

## 1. Introduction

It is well known that breastfeeding is the “gold standard” for infant and young child feeding [1,2]. Its promotion is crucial to achieving the Sustainable Development Goals and is essential to support planetary health [3,4,5]. A study published in the Lancet confirms that breastfeeding can save 823,000 infant lives each year and add USD 302 billion to the global economy [2]. Therefore, breastfeeding is a healthy, economical, and sustainable practice that must be promoted by various healthcare institutions and political entities, ensuring it is safe [6,7,8]. However, it is important to note that there are certain situations in which breastfeeding is contraindicated [9,10]. In promoting this practice, the nutritional status of mothers must be taken into account, since breastfeeding increases the total energy requirement by 400–500 kcal/day. Factors such as the duration and intensity of breastfeeding can have an impact on the mother’s nutritional status [11,12]. Therefore, it is necessary to attend to the diet of pregnant and lactating mothers, ensuring their optimal nutritional status and well-being, as well as the quality and safety of human milk, which will be the primary and/or exclusive sustenance of children from 0 to 6 months of age [13,14,15,16]. Few studies have considered the impact of breastfeeding on maternal nutritional status. Rather, the focus has been on the influence of maternal nutritional status on the composition of breast milk [17,18]. From these studies, it is known that the most common nutrient deficiencies in human milk are the result of maternal dietary deficiencies, usually related to water-soluble vitamins, thiamine, riboflavin, and vitamins B6 and B12. Other studies show that maternal malnutrition can also impair mammary gland function and the normal transport processes involved in the transfer of micronutrients to milk [19]. The CastelLact Project represents a groundbreaking initiative aimed at comprehensively analyzing the nutritional status and dietary patterns of pregnant and lactating mothers, alongside an unprecedented examination of breast milk composition in a specific region of Spain. This project stands as a vital contribution to the understanding of maternal and infant nutrition within this population, addressing a critical gap in regional nutritional data.

## 2. Materials and Methods

To achieve the proposed objective, a descriptive study of the diet quality of pregnant and lactating women in Castellon (Spain) was designed.

### 2.1. Sample Selection

Convenience sampling was carried out, and the inclusion criteria for study participants were pregnant women from southeast Spain who planned to breastfeed their babies and voluntarily agreed to provide the data requested in the study, as well as donate a sample of their mature breast milk following the indicated protocol. Finally, the sample consisted of 26 women with a median age of 32.3 years. Approval to conduct this study was obtained from the Ethics Committee of Human Research of the University of Valencia with procedure number H1493469177420 and approved on 8 May 2017. All participating mothers were informed in writing of the purpose and methodology of the study, and they signed an informed consent form. After completion of the study, a report with the obtained results was provided to them.

### 2.2. Data Collection

This research used quantitative, qualitative, and mixed data collection techniques to obtain information about the social and clinical–obstetric history of participating mothers, as well as dietary and anthropometric data. This information is essential to understanding the factors that can affect breastfeeding and to developing effective strategies to improve its practice. Information on social and clinical–obstetric history was collected during acceptance into the study, and after delivery, information related to gestational clinic and delivery type was collected through an in-person interview. Maternal anthropometric data were evaluated during pregnancy and measured using a SECA 220 stadiometer (with an accuracy of ±1 mm) for height and a TANITA BF-350 scale for weight with an accuracy of ±0.1 kg, following a protocol established by the International Society for Anthropometry Applied to Sport and Health (ISAnASHe) [20]. The weight gain of each mother throughout her pregnancy was calculated and compared with the recommended weight gain limits for pregnant women based on their pre-pregnancy body mass index (BMI) [21]. BMI calculation was established with usual weight and with weight after 2 weeks postpartum. To collect dietary data and eating habits, training sessions were conducted for participants. Three tools were used: a questionnaire on maternal dietary habits during pregnancy, a self-filled Food Frequency Questionnaire (FFQ) by each mother during the final stages of pregnancy, and a three-day dietary record (two workdays and a holiday) for the mother [22]. Conversion of food consumption into energy and nutrient intakes was performed using the DIAL program version 1.10 (Alce Ingeniería SA, Madrid, Spain, http://www.alceingenieria.net/nutricion.htm accessed on 21 May 2022). Furthermore, Spanish and European dietary reference intakes were taken into consideration [23,24,25,26,27].

### 2.3. Analysis of Breast Milk

**Figure 1:**
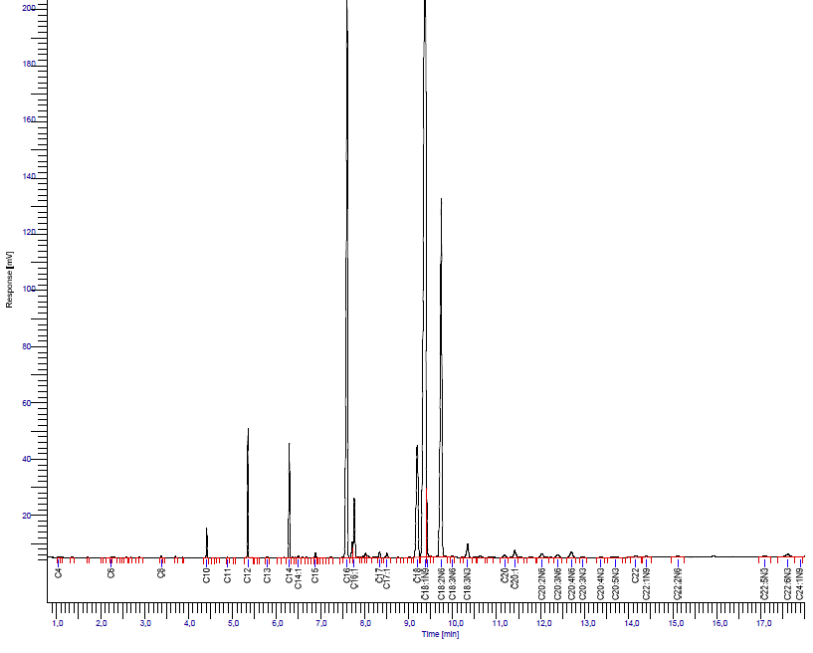
Gas–liquid chromatogram of fatty acid methyl esters.

Breast milk samples were assessed, in triplicate, for specific macronutrients (proteins, carbohydrates, fats, polyunsaturated fatty acids—PUFAs) and micronutrients (calcium, magnesium, selenium, zinc). The Association of Official Analytical Collaboration (AOAC) procedure [28] was used to analyze the moisture, ash and protein. Moisture was obtained by the gravimetric method, where 3 g of each sample was heated at 105 °C until reaching constant weight. Samples that had been calcinated in a muffle at 550 °C for 5 h were used to calculate the ash and protein analysis was carried out by the Kjeldahl procedure. The method of Folch et al. [29] was used to determine lipids with chloroform and methanol (2:1, *v*/*v*) and the extracts evaporated to dryness under nitrogen. Fatty acid methyl esters (FAMEs) were prepared through transesterification with boron trifluoride in methanol. Separation and identification of fatty acids were conducted using an Agilent 7890 gas chromatograph (GC) equipped with a flame ionization detector (FID) and an SP-2560 capillary GC column (100 m × 0.25 mm × 0.20 µm; Sigma-Aldrich Co., St. Louis, MO, USA). The column was calibrated against a standard mixture containing thirty-seven FA methyl esters with carbon chain lengths ranging from four to twenty-four (Supelco 37 Component Fame Mix; Supelco: Bellefonte, PA, USA). For the GC-FID analysis, the sample injection volume was set at 1 µL, with nitrogen as the carrier gas flowing at 1.15 mL/min, using a split ratio of 50:1, and constant flow control. The injector and detector temperatures were maintained at 225 °C and 285 °C, respectively. The oven temperature program started at 120 °C for the first 5 min, followed by an increase of 3 °C per minute to 210 °C, maintained for 3 min, then increased by 1 °C per minute to 230 °C, and held for 7 min. An aliquot of the resulting methyl esters was transferred into an autosampler vial for GC-FID analysis. Identification of FAMEs was achieved by comparing their relative retention times with those of authentic standards, and the quantification was performed by measuring the peak areas electronically. Each sample’s fatty acids were quantified as percentages of the total area under the fatty acid peaks and reported as a percentage of total fatty acids [30]. An example of GC separation of a human milk sample is depicted in Figure 1. According to Terra et al. [31], carbohydrate concentrations were determined by the difference between the total sample (100%) and the concentrations of the macronutrients content (protein, fat, moisture and ash). Gross total energy content was calculated as: Energy = proteins × 4 + fat × 9 + carbohydrates × 4 according to the Atwater general factor system. Lactose content was analyzed using a Bio-Flow^®^-4 (Oji Scientific Instruments, Amagasaki-shi, Hyogo, Japan) based on amperometric-enzymatic methods. Inductively coupled plasma atomic emission spectrometry (ICP-AES) measurements were carried out using a sequential plasma spectrometer ICPS-7500 system (Shimadzu Corporation, Kyoto, Japan) for simultaneous determination of calcium in breast milk. For preparation for the analysis, 10 mL aliquots of the whole milk samples were dried at 450 °C for 2 h in ceramic evaporating dishes, and then dissolved in 10 mL of distilled deionized water supplemented with 2 mL of 6 mol/L HCl. One milliliter of this was transferred into a 10 mL volumetric flask and the flask was filled with Milli-Q water. To determine the Ca contents of the sample, 10 mL of the last solution was transferred into a 100 mL volumetric flask and the flask was filled with Milli-Q water. The plasma source used for spectrophotometry was 99.998% argon, and the wavelength used for analysis of Ca was 422.673 nm. The calibration was performed using a blank and two standard concentrations for each element measured. The optimal operation conditions for ICP-AES analysis of Ca were the following: power, 1.2 kW; carrier gas flow rate, 0.7 L/min; plasma gas flow rate, 1.2 L/min; cooling gas flow rate, 14.0 L/min [32].

### 2.4. Statistical Data Processing

Statistical analysis was performed using IBM^®^ SPSS^®^ Statistics version 27 software (IBM Corp., Armonk, New York, NY, USA). Descriptive statistics were used to analyze the characteristics of the mothers and their dietary intake. Median, mean, standard deviation, maximum, and minimum values were calculated for quantitative variables, while frequency analysis was performed for qualitative variables, with percentages reported.

## 3. Results

### 3.1. Description of the Sociodemographic Characteristics of the Sample

The study sample consisted of 26 women aged between 25 and 40 years with a median age of 32.3 years. Of the sample, 38.50% (n = 10) had a medium education level and 61.50% (n = 16) had a higher education level. More than half of the participants (76.9%; n = 20) lived in urban areas as their habitual residence, while 23.10% (n = 6) lived in rural areas. Regarding previous pregnancies, the sample consisted of women who had between one and three pregnancies in their lifetime. For 50% of the mothers (n = 13), this was their first pregnancy. Of the mothers who had already had a previous pregnancy (n = 13), 84.6% (n = 13) had opted for breastfeeding previously.

### 3.2. Anthropometric and Clinical–Obstetric Information of the Mothers

**Figure 2:**
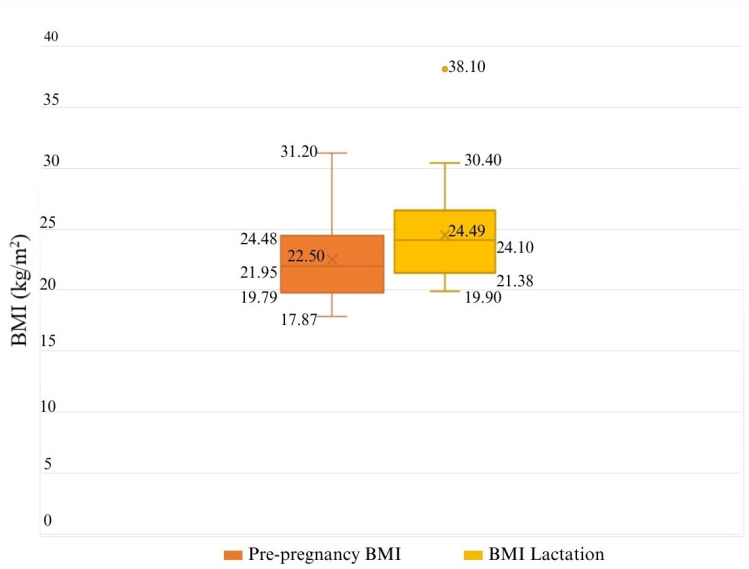
Anthropometric variations of mothers: evolution of body mass index before and after pregnancy.

The height of the mothers ranged from 1.59 m to 1.70 m, with a mean of 1.64 m ± 0.04 SD. The pre-pregnancy weight of the mothers ranged from 48 to 78 kg, with a mean of 60.30 kg ± 9.46 SD. The participants’ pre-pregnancy BMI ranged from 17.9 kg/m^2^ to 31.2 kg/m^2^ (Figure 2). The weight gain during pregnancy ranged from 6.5 kg to 20.3 kg. The mean weight gain during pregnancy was 12.8 kg ± 3.5 SD (Figure 2). The mean BMI varied from 22.5 kg/m^2^ ± 3.2 SD pre-pregnancy to 24.5 kg/m^2^ ± 3.9 SD during lactation, and an outlier value of 38.10 kg/m^2^ was observed. Of the sample, 53.8% engaged in regular physical activity, while the rest engaged in physical activity occasionally.

Out of the 26 participants, 3.8% (n = 1) had diabetes mellitus, 3.8% (n = 1) had hyperthyroidism, and 7.69% (n = 2) had hypothyroidism. The remaining participants reported no clinical conditions during pregnancy. Regarding the type of delivery, 65.4% (n = 17) of the cases were natural births, while 34.6% (n = 9) were by cesarean section. The majority of participants (57.69%; n = 15) reported lactation complications, with 38.5% (n = 10) experiencing nipple cracks and 19.2% (n = 5) suffering from mastitis.

### 3.3. Dietary and Eating Habits Study during Pregnancy and Lactation

**Figure 3:**
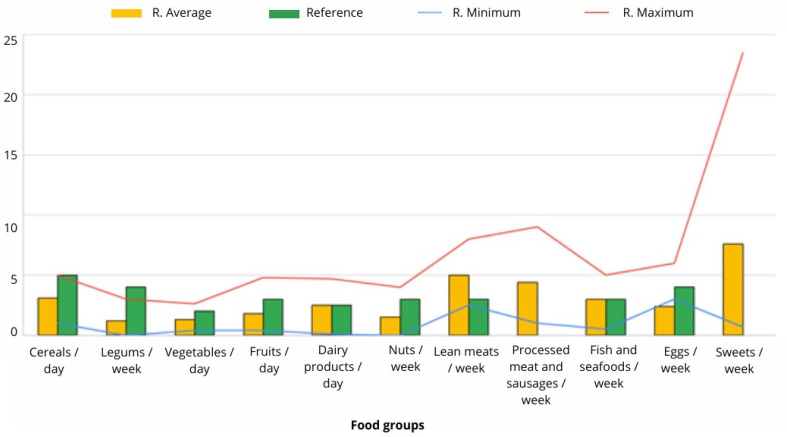
Frequency of consumption of the different food groups by mothers during pregnancy according to recommendations [24,25,26,27]. R: Ration.

**Figure 4:**
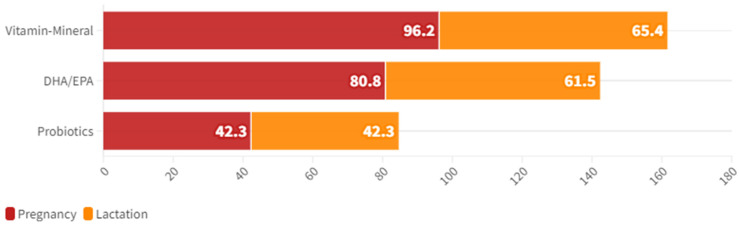
Oral supplementation during pregnancy and lactation.

During pregnancy, 53.8% (n = 14) of the sample reported consuming five meals per day, with a minimum of three meals per day reported by 7.7% (n = 2) and a maximum of six meals per day reported by 11.5% (n = 3) of women. The rest reported consuming four meals per day as a usual practice. The most common cooking technique was grilling (92.3%; n = 24), followed by baking (76.9%; n = 20) and boiling (69.2%; n = 18). Frying was the least common technique used regularly, with only 15.4% (n = 4) reporting its use. The frequency of food consumption during pregnancy is presented in Figure 3, and the oral supplementation is presented in Figure 4.

**Figure 5:**
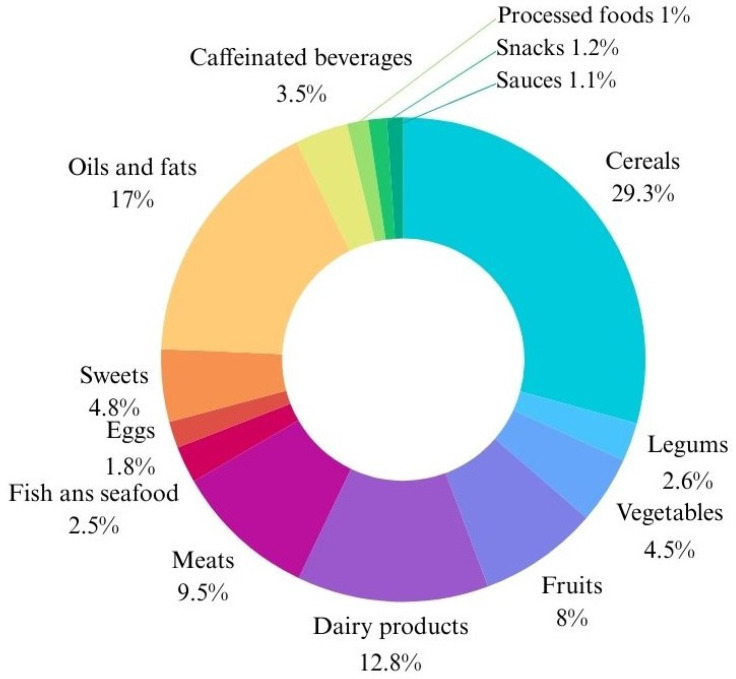
Distribution of the percentage of total caloric value by food group in the diet of nursing mothers.

The nutritional study of the diet of mothers during lactation reflects that the average energy intake was 2575.88 kcal/day ± 730.59 SD. Fifteen percent of the total energy intake throughout the day came from proteins, 45% from carbohydrates, and 40% from lipids. Table 1 and Table 2 and Figure 5 show further nutritional information.

In Table 3, it can be observed that 100% (n = 26) of the lactating mothers consumed at least four meals a day and that the recommended energy percentage for each of the daily meals for this group was not met.

### 3.4. Results of the Breast Milk Analysis

The analysis of breast milk samples (Table 4), by gas chromatography, revealed a wide range in the nutritional composition. The average energy content was 66.08 kcal, aligning closely with standard references.

The fat composition showed significant variability, with Gerber’s fat percentage averaging 3.82%, and saturated fatty acids (FSA) comprising 43.71% of total fatty acids. Polyunsaturated fatty acids (PUFA) averaged 23.36%, while monounsaturated fatty acids (MUFA) were at 32.96%. Specific fatty acids such as C:10:0 and C:12:0 had lower concentrations compared to reference values. Protein content averaged 1.05%, and carbohydrate content was 7.13%, both slightly below recommended levels. Lactose content averaged 6.48%. The dry extract percentage was 11.83%, and humidity was 88.17%, both close to standard values. Ash content was lower than reference levels, averaging 0.14%. The calcium content was 249.81 mg/L, which is below the recommended range. The cryoscopic point averaged −0.56 °C, indicating consistency in milk composition. Overall, while the nutritional composition of breast milk showed some deviations from recommended values, particularly in fat and calcium content, it generally aligns closely with established standards. These findings highlight the importance of monitoring and optimizing maternal nutrition to ensure optimal breast milk quality.

## 4. Discussion

This study assessed the nutritional status of a sample of pregnant mothers who subsequently went on to lactation, and examined their habits and dietary intake to evaluate the adequacy of their diets based on established recommendations for this population group. During pregnancy and lactation, there is an increased requirement for almost all nutrients compared to the needs of a woman of the same age [34,35].

Anthropometric values, such as body mass index (BMI), were monitored during pregnancy and lactation to ensure optimal weight gain and avoid obstetric complications and postpartum health problems [36]. The study found that the pregnant and lactating women generally had normal BMI values, although a few cases of overweight and obesity were detected. The weight gain during pregnancy was optimal according to the recommendations, but one case of diabetes and three cases of thyroid disorders were detected, which could be due to hormonal imbalances during pregnancy [37].

The dietary habits of the pregnant and lactating mothers were found to be inadequate, with food choices deviating significantly from the Mediterranean diet [38]. Other studies have also reported deficiencies in the intake of fruits, vegetables, legumes, nuts, eggs, and fish, as well as excess consumption of processed meats, pastries, and sweets [39,40,41]. The trend of moving away from the Mediterranean diet is not limited to this population group, but is a widespread phenomenon among different age and gender groups in the southeast of Spain and in other Mediterranean countries, as the influence of the Western dietary pattern, which is far from healthy and sustainable, is observed [42,43,44]. The results of this study and those of other studies reveal that mothers’ high nutritional requirements during lactation are often overlooked after childbirth, which can ultimately affect the variability of certain micronutrients and the lipid profile of breast milk, negatively impacting the present and future health of mothers and infants and possibly having negative repercussions for the economy and the environment [45].

The average energy intake during lactation was close to the recommendations for this group, reaching 95%, and was slightly higher than values reported in other studies [45,46,47,48]. However, the distribution of energy between macronutrients and micronutrients was not optimal, as reported in previous studies [46]. The present study found an inadequate energy distribution with an excess of lipids. Factors such as poor nutritional knowledge, lack of time for meal preparation, and dietary restrictions can contribute to inadequate energy intake. This can result in a diet that is disproportionately high in lipids, as mothers may rely on convenient, high-fat foods. The caloric distribution between meals was also imbalanced compared to the reference distribution [49]. Overall, it is crucial for lactating mothers to receive proper nutritional counseling and support to ensure a balanced intake of macronutrients, aligning with their increased energy needs during lactation

The lipid profile of the maternal diet during lactation differed from the recommendations, and the intake of saturated fatty acids (SFA) was found to be doubled in the diet (as shown in Table 1), which could affect the lipid profile of human milk. This excess intake of saturated fat is in line with what has been observed in other studies [47]. Therefore, this group may be susceptible to a possible energy/nutritional deficiency. Dietary habits are key to supplementing the risk of subclinical malnutrition in folic acid, iodine, vitamin C, calcium, or iron due to increased needs during pregnancy or lactation [50,51,52]. From their study, it is derived that B-group vitamins, except for folic acid, pantothenic acid, and biotin, showed values much higher than the Dietary Reference Intake (DRI). Among the fat-soluble vitamins, elevated values were observed in vitamin K and slightly elevated in vitamin A. However, vitamin E and vitamin D were especially deficient. The explanation for this could be due to the lack of consideration of vitamin supplementation in milk after skimming processes, which leads to an underestimation of the content of these vitamins in semi-skimmed or skimmed milk, which also represent the majority consumption. In addition, in the southeast of Spain, with good weather, the endogenous synthesis of active vitamin D from its precursors can also be expected by solar exposure. Compared to Arija et al. [53], higher values of vitamin D, E, C, B1, B2, and B6 were found in the diets of lactating women in the present study, and practically similar values of folic acid and vitamin B12 were found.

Regarding mineral intake, except for calcium, iodine, and zinc, the rest of the minerals were supplied in values adjusted to the DRI or even higher [25,27]. Other studies also detected deficiencies in these minerals [54,55,56]. On the other hand, the calcium deficiency is in line with the deficiency in vitamin D, and may be related to a consumption of dairy products that is minimally adjusted to recommendations [25]. In the present work, it was also found that pregnant and lactating women took some supplements. Generally, supplementation is preceded by medical indication to ensure nutrients during pregnancy and lactation [50,57,58,59,60,61]. There was a decrease of almost 30% in the consumption of vitamin/mineral supplements and a 20% decrease in the consumption of supplements with Docosahexaenoic Acid (DHA) and/or Eicosapentaenoic Acid (EPA) between pregnancy and lactation, although the consumption of probiotic foods remained constant. This leads to the belief that requirements during lactation are undervalued compared to pregnancy.

The prevalence of clinical conditions during pregnancy in this study was relatively low. However, lactation complications were more frequent, affecting over half of the participants. Nipple cracks and mastitis are two of the most common breastfeeding problems reported by postpartum women. These complications can cause significant discomfort, pain, and even interfere with the continuation of breastfeeding. Thus, it is essential to provide support and education to new mothers to help prevent and manage these complications. Overall, the results suggest that there is a need for greater awareness of and education on proper nutrition and dietary habits during pregnancy and lactation [61,62,63]. Additionally, healthcare professionals should provide more support and guidance to help mothers overcome any difficulties they may encounter during the breastfeeding process [61,62,63,64].

In order to ensure maternal–child health and planetary health, and to ensure healthy eating for pregnant and lactating women, the literature recommends adopting a personalized approach to nutritional counseling, taking into account the access of pregnant and breastfeeding women to food, socioeconomic situation, race, ethnic origin, and cultural food options, as well as BMI [1,2,3,4,5]. In addition, paying attention to individual health-disease states to assess whether there are situations in which breastfeeding is contraindicated is a priority [9,64]. It is essential to reinforce awareness and the importance of maternal diet not only during pregnancy but also during lactation, as this study and other research show that malnutrition may exist in this group [5,6,9]. Ensuring adequate breastfeeding is a priority for public and planetary health, and for that, attention must be paid to women’s nutritional status and other lifestyles that directly or indirectly affect the achievement of this goal [2,5,6].

This study has several limitations that should be considered when interpreting the results. First, the sample size was relatively small (n = 26), which may limit the generalizability of our findings. The participants were from diverse backgrounds, including both urban and rural areas, and varied in educational levels and dietary habits. Additionally, some participants were taking vitamin supplements while others were not, which could have influenced the nutritional assessments. These variations may introduce confounding factors that were not fully controlled in the study. Furthermore, the extended period required for data collection was due to recruitment challenges and the time needed for protocol approval, which was granted in 2017. These factors may have also contributed to the limited sample size. Future studies with larger and more homogeneous populations are needed to validate these findings and provide more comprehensive insights.

## 5. Conclusions

In conclusion, 84.7% of the participating mothers in this study exhibited anthropometric values within the normal range (BMI 19.0–24.9 kg/m^2^) and gained weight during pregnancy in accordance with established recommendations (12.8 ± 3.5 kg). However, the dietary intake of mothers during pregnancy was deficient in fruits, vegetables, legumes, nuts, and cereals, indicating a departure from recommended consumption patterns. Similarly, the calibrated diet of lactating mothers only partially adhered to established nutritional goals, with an excessive intake of saturated fats at the expense of carbohydrates. Furthermore, the diet exhibited deficiencies in key vitamins and minerals, including calcium, iodine, vitamin D, E, and folic acid. Therefore, promoting and encouraging proper dietary practices during pregnancy and lactation is crucial to ensuring the health of both mothers and their newborns, preventing the development of chronic diseases, and supporting planetary health.

## Figures and Tables

**Table 1:** Daily intake of energy, macronutrients, and lipid profile of lactating mothers’ diets, obtained from dietary records.

Parameters	Minimum–Maximum	Average ± SD	Spanish Recommendation *	European Recommendations ^†^
Energy (kcal)	1731.00–5108.00	2575.88 ± 730.59	2700	2647
Proteins (g)	57.40–204.00	96.81 ± 33.93	1.3 g/kg/d: 78 g	0.83 g/kg/d + 19 g
% protein kcal	8.65–20.46	15.01 ± 2.71	10–15%	
Carbohydrates (CH) (g)	145.00–591.00	260.41 ± 90.92		
% kcal CH	29.58–52.13	40.22 ± 6.12	50–55%	45–60%
Fiber (g)	14.60–45.50	23.64 ± 6.48	>25	
Lipids (g)	73.00–221.00	123.46 ± 32.42		
% lipids kcal	34.18–54.40	43.51 ± 5.47	30–35%	20–35%
SFA (g)	21.70–70.20	37.16 ± 10.43		
% VCT SFA	8.68–17.32	13.13 ± 2.16	≤7–8%	Minimum
MUFA (g)	35.30–103.00	58.22 ± 15.82		
% VCT MUFA	15.50–26.66	20.62 ± 3.68	20%	
PUFA (g)	7.10–35.00	18.78 ± 7.15		
% VCT PUFA	3.69–13.74	6.55 ± 3.69	5%	
Cholesterol (mg)	84.70–702.00	331.38 ± 140.43	<300	
C14:0 (g)	0.49–5.60	2.83 ± 1.17		
C16:0 (g)	12.00–35.80	20.39 ± 5.51		
C18:0 (g)	4.40–14.40	8.08 ± 2.48		
C16:1 (g)	0.59–2.80	1.55 ± 0.60		
C18:1 (g)	33.80–93.50	53.33 ± 13.88		
C18:2 (g) (AL n-6)	5.60–32.20	16.08 ± 6.59	13 g/d	4%
C18:3 (g) (ALA n-3)	0.61–3.00	1.54 ± 0.55	1.3 g/d	0.5%
C20:4 (g)	0.01–0.77	0.14 ± 0.15		
C20:5 (g) (EPA)	0.00–0.73	0.10 ± 0.15		
C22:5 (g)	0.00–0.08	0.03 ± 0.02		
C22:6 (g)(DHA)	0.00–0.97	0.21 ± 0.21	200–300 mg	350–450 mg
Trans FA	0.00–2.47	0.57 ± 0.57	Minimum	Minimum
%VCT Trans FA	0.00–0.72	0.21 ± 0.19	<1%VCT	
(PUFA+MUFA)/SFA	1.45–2.96	2.10 ± 0.37	≥2	
PUFA/SFA	0.35–0.79	0.50 ± 0.13	≥0.5	

* References from [24,25,26]. **^†^** Reference [27]. %kcal CH: Percentage of kilocalories from Carbohydrates; % lipids kcal: Percentage of kilocalories from Lipids; SFA (g): Saturated Fatty Acids (grams); % VCT SFA: Percentage of Total Caloric Value from Saturated Fatty Acids; MUFA (g): Monounsaturated Fatty Acids (grams); % VCT MUFA: Percentage of Total Caloric Value from Monounsaturated Fatty Acids; PUFA (g): Polyunsaturated Fatty Acids (grams); % VCT PUFA: Percentage of Total Caloric Value from Polyunsaturated Fatty Acids; C14:0 (g): Myristic Acid (grams); C16:0 (g): Palmitic Acid (grams); C18:0 (g): Stearic Acid (grams); C16:1 (g): Palmitoleic Acid (grams); C18:1 (g): Oleic Acid (grams); C18:2 (g) (AL n-6): Linoleic Acid (omega-6) (grams); C18:3 (g) (ALA n-3): Alpha-Linolenic Acid (omega-3) (grams); C20:4 (g): Arachidonic Acid (grams); C20:5 (g) (EPA): Eicosapentaenoic Acid (grams); C22:5 (g): Docosapentaenoic Acid (grams); C22:6 (g) (DHA): Docosahexaenoic Acid (grams); Trans FA: Trans Fatty Acids; %VCT Trans FA: Percentage of Total Caloric Value from Trans Fatty Acids; (PUFA+MUFA)/SFA: Ratio of (Polyunsaturated Fatty Acids + Monounsaturated Fatty Acids) to Saturated Fatty Acids; PUFA/SFA: Ratio of Polyunsaturated Fatty Acids to Saturated Fatty Acids; SD: Standard Deviation.

**Table 2:** Vitamin and mineral content of the diet of nursing mothers compared to recommendations.

Nutrients		Minimum–Maximum	Average ± SD	Spanish Recommendation *	European Recommendation ^†^
Vitamins	Vit. B1 (mg)	0.94–5.20	1.81 ± 0.89	1.4	1
	Vit. B2 (mg)	1.30–8.00	2.55 ± 1.35	1.7	2
	Niacin (mg)	24.20–107.00	40.17 ± 16.96	16	16
	Vit. B6 (mg)	1.20–7.50	2.40 ± 1.19	1.6	1.7
	Vit. B12 (µg)	0.86–15.40	5.72 ± 2.80	2.6	5
	Folic acid (µg)	2.16–790.00	274.23 ± 130.25	400	500
	Vit. C (mg)	47.20–429.00	127.69 ± 74.14	100	155
	Vit. A (µg)	552.00–2095.00	1042.78 ± 369.86	950	1300
	Vit. D (µg)	0.31–13.90	3.29 ± 2.87	10	15
	Vit. E (mg)	6.40–29.90	12.84 ± 5.81	19	11
	Vit. K (µg)	69.40–455.00	136.96 ± 74.46	90	70
	Ac. Pantothenic (mg)	3.00–12.80	5.88 ± 1.94	7	7
	Biotin (µg)	15.20–63.70	31.98 ± 11.63	35	45
Minerals	Calcium (mg)	508.00–2111.00	1062.47 ± 348.08	1200	950–1000
	Iron (mg)	9.80–40.80	16.09 ± 5.81	15	16
	Iodine (µg)	42.80–288.00	116.22 ± 55.32	200	200
	Magnesium (mg)	203.00–816.00	363.63 ± 127.84	360	300
	Zinc (mg)	7.00–21.40	11.33 ± 3.27	12	10.4–15.6
	Sodium (mg)	1374.00–4442.00	2311.47 ± 738.78	1500	2000
	Potassium (mg)	1435.00–7208.00	3430.76 ± 1067.95	3100	4000
	Phosphorus (mg)	971.00–3117.00	1601.68 ± 473.99	990	550
	Selenium (µg)	45.20–245.00	118.90 ± 42.87	70	85
	Copper (mg)	0.68–3.70	1.60 ± 0.66	1.4	1.5
	Chromium (µg)	10.10–98.50	46.98 ± 19.70	45	-
	Chlorine (mg)	1174.00–7973.00	2128.85 ± 1285.69	2300	3100
	Manganese (mg)	1.80–9.10	3.66 ± 1.71	2.6	3

* References from [24,25,26]. **^†^** Reference [27]. SD: Standard Deviation.

**Table 3:** Energy distribution (in calories) of the diet of nursing mothers in the different daily intakes compared with the recommendations.

Intakes	Minimum–Maximum	Average ± SD	Recommendation of Energy Distribution (24)	Caloric Distribution by Intake and Compliance
Breakfast	187–2034	434.87 ± 368.61	25%	16%
Morning snack	0–700	170.83 ± 177.38	10%	6%
Lunch	569–1466	934.22 ± 261.39	30%	35%
Afternoon snack	144–736	331.22 ± 147.19	10%	13%
Dinner	385–1278	745.13 ± 233.83	25%	29%
Night snack	0–144	20.00 ± 40.46		

SD: Standard Deviation.

**Table 4:** Nutritional composition of a sample of human milk.

Composition	Minimum–Maximum	Average ± SD	Reference [33]
Energy (Kcal)	20–100	66.08 ± 15.26	64–72
G. Gerber (%)	1.5–7.6	3.82 ± 1.60	3.8–4.2
FSA (g/100 g/%FA)	0.78–2.78	1.6/43.71 ± 0.67	38.5–41.8%
PUFA (g/100 g/%FA)	0.17–2.08	0.86/23.36 ± 0.54	15.5–22.8%
MUFA (g/100 g/%FA)	0.31–2.68	1.21/32.96 ± 0.74	39.1–43.0%
C4:0	0.05–0.10	0.05 ± 0.010	-
C:5:0	0.05–0.05	0.05 ± 0.00	
C:6:0	0.05–0.10	0.05 ± 0.01	0.02–0.58
C:7:0	0.05–0.05	0.05 ± 0.00	
C:8:0	0.05–0.10	0.07 ± 0.03	0.13–0.28
C:10:0	0.40–1.90	0.80 ± 0.31	1.10–1.77
C:11:0	0.05–0.05	0.05 ± 0.00	0.03–0.05
C:12:0	1.51–7.98	3.61 ± 1.46	5.15–8.60
C:13:0	0.05–0.05	0.05 ± 0.00	0.02–0.04
C:14:0	1.90–9.00	4.92 ± 1.73	4.34–7.00
C:15:0	0.10–0.60	0.27 ± 0.12	0.15–0.30
C:16:0	17.30–31.20	23.85 ± 3.39	15.00–21.26
C:17:0	0.20–0.50	0.31 ± 0.09	0.25–0.30
C:18:0	5.10–10.90	7.36 ± 1.43	5.05–7.59
FSA (g/100 g)	0.78–2.78	1.60 ± 0.67	
C:12:1	0.05–0.05	0.05 ± 0.00	
C:14:1	0.05–0.30	0.11 ± 0.05	
C:18:1	30.40–48.70	38.60 ± 5.13	34.61–39.11
MUFA (g/100 g)	0.31–2.68	1.21 ± 0.74	
C:18:3	0.40–1.30	0.76 ± 0.24	0.60–1.01
C:20:3	0.05–0.50	0.23 ± 0.16	0.03–0.56
C:22:3	0.05–0.05	0.05 ± 0.00	
C:22:6	0.05–0.70	0.34 ± 0.21	0.27–0.37
PUFA (g/100 g)	0.17–2.08	0.86 ± 0.54	
Protein (%)	0.20–1.5	1.05 ± 0.30	0.9–1.1
Carbohydrates (%)	2.30–10.10	7.13 ± 2.01	7.7–7.9
Lactose (%)	2.1–8.90	6.48 ± 1.99	7–7.3
Extracto seco (%)	4.8–15.80	11.83–2.29	12–13
Humidity (%)	84.20–95.20	88.17 ± 2.29	87–88
Ashes (%)	0.05–0.20	0.14 ± 0.07	0.2
Calcium (mg/l)	140.00–334.00	249.81 ± 59.41	280–340
Cryoscopic Point (°C)	−0.70–−0.11	−0.56 ± 0.11	

FSA: (g): Saturated Fatty Acids (grams); MUFA: Monounsaturated Fatty Acids; PUFA: Polyunsaturated Fatty Acids; SD: Standard Deviation.

## Data Availability

The original contributions presented in the study are included in the article, further inquiries can be directed to the corresponding authors.
